# Veiga IPA, Caldeira TCM, Soares MM, Sousa TM, Silva LES, Claro RM. Fruit and vegetable consumption among Brazilian adults: trends from 2008 to 2023. Cad Saúde Pública 2025; 41(1):e00032424.

**DOI:** 10.1590/0102-311XER032424

**Published:** 2025-02-28

Where it reads:

Vigitel conducted 697,549 interviews with adult individuals (≥ 18 years) from 2008 to 2023. Over the entire period, a decrease was observed in the proportion of young adults (18-24 years), declining from 17.9% to 12.9% (-0.33pp/year), while an increase in the proportion of individuals aged 55-64 years was observed, rising from 10.4% to 13.9% (0.25pp/year). Regarding schooling level, there was a decrease in individuals with 0 to 8 years of schooling, from 43.7% to 25.8% (-1.28pp/year), and an increase in adults with 12 years of schooling or more, from 21.6% to 32.9% (0.78pp/year) (Table 1).

The prevalence of regular fruit and vegetable consumption (≥ 5 days/week) remained stable from 2008 to 2023, with an average of 34.1% for the entire population and among all sociodemographic groups. In the initial period (2008-2014), regular fruit and vegetable consumption increased from 33% to 36.5% (0.71pp/year) for the entire population and almost all sociodemographic groups, except for adults aged 25 to 34 years. The highest increase during this period was observed among women (0.75pp/year), individuals aged 65 and more (0.83pp/year), and adults with 0 to 8 years of schooling (0.69pp/year). Conversely, the analysis of the most recent period (2015-2023) showed a decrease in the prevalence of fruit and vegetable consumption for the entire population, from 37.6% to 31.9% (-0.56pp/year), particularly among men (-0.70pp/year), individuals aged 25-34 years (-0.84pp/year) and with more than 12 years of schooling (-0.96pp/year) (Table 2 and Supplementary Material - Table S1: https://cadernos.ensp.fiocruz.br/static//arquivo/suppl-e00032424_6302.pdf).

It should read:

Vigitel conducted 697,549 interviews with adult individuals (≥ 18 years) from 2008 to 2023. Over the entire period, a decrease was observed in the proportion of young adults (18-24 years), declining from 17.9% to 12.9% (-0.37pp/year), while an increase in the proportion of individuals aged 55-64 years was observed, rising from 10.4% to 13.9% (0.24pp/year). Regarding schooling level, there was a decrease in individuals with 0 to 8 years of schooling, from 43.7% to 25.8% (-1.27pp/year), and an increase in adults with 12 years of schooling or more, from 21.6% to 32.9% (0.87pp/year) (Table 1).

The prevalence of regular fruit and vegetable consumption (≥ 5 days/week) remained stable from 2008 to 2023, with an average of 34.1% for the entire population and among all sociodemographic groups. In the initial period (2008-2014), regular fruit and vegetable consumption increased from 33% to 36.5% (0.71pp/year) for the entire population and almost all sociodemographic groups, except for adults aged 25 to 34 years. The highest increase during this period was observed among women (0.75pp/year), individuals aged 65 and more (0.83pp/year), and adults with 0 to 8 years of schooling (0.69pp/year). Conversely, the analysis of the most recent period (2015-2023) showed a decrease in the prevalence of fruit and vegetable consumption for the entire population, from 37.6% to 31.9% (-0.58pp/year), particularly among women (-0.54pp/year), individuals aged 35-54 years (-0.94pp/year) and with more than 12 years of schooling (-0.99pp/year) (Table 2 and Supplementary Material - Table S1: https://cadernos.ensp.fiocruz.br/static//arquivo/suppl-e00032424_6302.pdf).

Where it reads:

**Table 1:** Distribution of the adult population (≥ 18 years) of Brazilian capitals and the Federal District according to sociodemographic characteristics. *Risk and Protective Factors Surveillance System for Chronic Noncomunicable Diseases Through Telephone Interview* (Vigitel), 2008- 2023 (n = 697,549).

Characteristics	2008 (%)	2014 (%)	2015 (%)	2023 (%)	Average increment (2008-2014) (pp/year) *	Average increment (2015-2023) (pp/year) *	Average increment (2008-2023) (pp/year) *
Sex							
Male	46.1	46.1	46.0	46.1	-0.01 **	-0.01 **	-0.02 **
Female	53.9	53.9	54.0	53.9	0.01 **	0.01 **	0.02 **
Age (years)							
18-24	17.9	15.6	15.2	12.9	-0.36 **	-0.39 **	-0.33 **
25-34	25.4	25.3	25.2	24.9	-0.04 **	-0.03 ***	-0.05 **
35-44	20.4	19.6	19.4	18.3	-0.14 **	-0.13 **	-0.15 **
45-54	16.1	17.1	17.3	18.3	0.16 **	0.17 **	0.15 **
55-64	10.4	11.8	12.1	13.9	0.24 **	0.24 **	0.25 **
65 and more	9.8	10.6	10.8	11.8	0.14 **	0.14 **	0.14 **
Schooling (years of study)							
0-8	43.7	35.9	34.6	25.8	-1.30 **	-1.33 **	-1.28 **
9-11	34.7	38.1	38.1	41.3	0.30 **	0.59 ***	0.51 ***
12 and more	21.6	25.9	27.3	32.9	1.00 **	0.78 **	0.78 ***

* Corresponding to the Prais-Winsten regression coefficient of the indicator value over the survey year;

** p < 0.000;

*** p between 0.05-0.001.

It should read:

**Table 1:** Distribution of the adult population (≥ 18 years) of Brazilian capitals and the Federal District according to sociodemographic characteristics. *Risk and Protective Factors Surveillance System for Chronic Noncomunicable Diseases Through Telephone Interview* (Vigitel), 2008- 2023 (n = 697,549).

Characteristics	2008 (%)	2014 (%)	2015 (%)	2023 (%)	Average increment (2008-2014) (pp/year) *	Average increment (2015-2023) (pp/year) *	Average increment (2008-2023) (pp/year) *
Sex							
Male	46.1	46.1	46.0	46.1	-0.01 **	-0.02 **	-0.01 **
Female	53.9	53.9	54.0	53.9	0.01 **	0.02 **	0.01 **
Age (years)							
18-24	17.9	15.6	15.2	12.9	-0.39 **	-0.34 **	-0.37 **
25-34	25.4	25.3	25.2	24.9	-0.03 ***	-0.05 ***	-0.04 **
35-44	20.4	19.6	19.4	18.3	-0.13 **	-0.15 **	-0.14 **
45-54	16.1	17.1	17.3	18.3	0.17 **	0.15 **	0.17 **
55-64	10.4	11.8	12.1	13.9	0.24 **	0.25 **	0.24 **
65 and more	9.8	10.6	10.8	11.8	0.14 **	0.14 **	0.14 **
Schooling (years of study)							
0-8	43.7	35.9	34.6	25.8	-1.33 **	-1.26 **	-1.27 **
9-11	34.7	38.1	38.1	41.3	0.59 ***	0.56 **	0.38 ***
12 and more	21.6	25.9	27.3	32.9	0.78 **	0.70 ***	0.87 **

* Corresponding to the Prais-Winsten regression coefficient of the indicator value over the survey year;

** p between < 0.05 and > 0.001;

*** p < 0.001.

Where it reads:

**Table 2:** Percentage of the adult population (≥ 18 years) that consumes fruits and vegetables on five or more days of the week (regular consumption) in Brazilian state capitals and in the Federal District, according to sociodemographic characteristics. *Risk and Protective Factors Surveillance System for Chronic Noncomunicable Diseases Through Telephone Interview* (Vigitel), 2008- 2023 (n = 697,549).

Characteristics	2008 (%)	2014 (%)	2015 (%)	2023 (%)	Average increment (2008-2014) (pp/year) *	Average increment (2015-2023) (pp/year) *	Average increment (2008-2023) (pp/year) *
Sex							
Male	26.4	29.4	31.3	27.9	0.67 **	-0.70 **	0.02
Female	38.6	42.5	43.1	35.3	0.75 **	-0.47 **	0.16
Age (years)							
18-24	24.6	27.5	29.3	26.0	0.44 **	-0.66 **	0.10
25-34	29.6	33.9	35.3	28.0	0.78	-0.84 **	0.15
35-44	31.7	33.9	35.7	27.5	0.50 **	-0.61 **	-0.02
45-54	37.0	38.7	39.2	34.0	0.75 **	-0.64 **	-0.05
55-64	40.7	44.6	44.6	37.4	0.76 **	-0.65	-0.07
65 and more	45.3	47.6	48.1	43.5	0.83 **	-0.35	-0.06
Schooling (years of study)							
0-8	29.5	32.4	33.0	28.1	0.69 **	-0.09	0.19
9-11	31.0	33.4	33.8	28.6	0.55 **	-0.89 ***	-0.19
12 and more	43.2	46.5	48.9	38.9	0.58 **	-0.96 **	-0.11
Total	33.0	36.5	37.6	31.9	0.71 **	-0.56 **	0.10

* Corresponding to the Prais-Winsten regression coefficient of the indicator value over the survey year;

** p between 0.05-0.001;

*** p < 0.000.

It should read:

**Table 2:** Percentage of the adult population (≥ 18 years) that consumes fruits and vegetables on five or more days of the week (regular consumption) in Brazilian state capitals and in the Federal District, according to sociodemographic characteristics. *Risk and Protective Factors Surveillance System for Chronic Noncomunicable Diseases Through Telephone Interview* (Vigitel), 2008- 2023 (n = 697,549).

Characteristics	2008 (%)	2014 (%)	2015 (%)	2023 (%)	Average increment (2008-2014) (pp/year) *	Average increment (2015-2023) (pp/year) *	Average increment (2008-2023) (pp/year) *
Sex							
Male	26.4	29.4	31.3	27.9	0.67 **	-0.48 **	0.03
Female	38.6	42.5	43.1	35.3	0.75 **	-0.54 **	-0.04
Age (years)							
18-24	24.6	27.5	29.3	26.0	0.44 **	-0.55 **	0.07
25-34	29.6	33.9	35.3	28.0	0.78	-0.82 **	0.02
35-44	31.7	33.9	35.7	27.5	0.50 **	-0.94 **	-0.23
45-54	37.0	38.7	39.2	34.0	0.75 **	-0.67 **	-0.16
55-64	40.7	44.6	44.6	37.4	0.76 **	-0.77 **	-0.21
65 and more	45.3	47.6	48.1	43.5	0.83 **	-0.35	-0.10
Schooling (years of study)							
0-8	29.5	32.4	33.0	28.1	0.69 **	-0.12	0.07
9-11	31.0	33.4	33.8	28.6	0.55 **	-0.75 **	-0.21
12 and more	43.2	46.5	48.9	38.9	0.58 **	-0.99 **	-0.27
Total	33.0	36.5	37.6	31.9	0.71 **	-0.58 **	-0.03

* Corresponding to the Prais-Winsten regression coefficient of the indicator value over the survey year;

** p < 0.001.

Where it reads:

**Table 3:** Percentage of the adult population (≥ 18 years) that consumes five or more daily servings of fruits and vegetables on five or more days of the week (recommended consumption) in Brazilian state capitals and in the Federal District, according to sociodemographic characteristics. *Risk and Protective Factors Surveillance System for Chronic Noncomunicable Diseases Through Telephone Interview* (Vigitel), 2008- 2023 (n = 697,549).

Characteristics	2008 (%)	2014 (%)	2015 (%)	2023 (%)	Average increment (2008-2014) (pp/year) *	Average increment (2015-2023) (pp/year) *	Average increment (2008-2023) (pp/year) *
Sex							
Male	15.8	19.3	21.0	19.3	0.71 **	-0.57 ***	0.11
Female	23.7	28.2	28.9	23.2	0.90 ***	-0.48 ***	0.23
Age (years)							
18-24	15.6	19.2	21.0	20.2	0.64 **	-0.63 ***	0.22
25-34	18.3	22.7	25.3	20.8	0.86 ***	-0.76 ***	0.27
35-44	19.4	23.4	24.2	18.2	0.68 ***	-0.40 ***	0.19
45-54	22.3	25.9	26.3	21.5	0.85 ***	-0.49 **	0.04
55-64	23.6	28.7	28.8	24.9	0.96 **	-0.46 ***	0.09
65 and more	26.3	27.8	27.3	24.8	0.57	-0.28	0.04
Schooling (years of study)							
0-8	16.9	20.2	20.1	17.1	0.69 ***	-0.23 **	0.14
9-11	19.6	22.5	23.2	19.5	0.71 ***	-0.79 **	-0.05
12 and more	27.1	31.9	34.6	27.2	0.77 ***	-0.73 ***	0.13
Total	20.0	24.1	25.2	21.4	0.81 ***	-0.52 **	0.17

* Corresponding to the Prais-Winsten regression coefficient of the indicator value over the survey year;

** p < 0.000;

*** p between 0.05-0.001.

It should read:

**Table 3:** Percentage of the adult population (≥ 18 years) that consumes five or more daily servings of fruits and vegetables on five or more days of the week (recommended consumption) in Brazilian state capitals and in the Federal District, according to sociodemographic characteristics. *Risk and Protective Factors Surveillance System for Chronic Noncomunicable Diseases Through Telephone Interview* (Vigitel), 2008- 2023 (n = 697,549).

Characteristics	2008 (%)	2014 (%)	2015 (%)	2023 (%)	Average increment (2008-2014) (pp/year) *	Average increment (2015-2023) (pp/year) *	Average increment (2008-2023) (pp/year) *
Sex							
Male	15.8	19.3	21.0	19.3	0.71 **	-0.31 ***	0.15
Female	23.7	28.2	28.9	23.2	0.90 ^#^	-0.68 **^,^ ***	0.11
Age (years)							
18-24	15.6	19.2	21.0	20.2	0.64 **	-0.26	0.24
25-34	18.3	22.7	25.3	20.8	0.86 **	-0.73 **	0.20
35-44	19.4	23.4	24.2	18.2	0.68 **	-0.72 **	-0.03
45-54	22.3	25.9	26.3	21.5	0.85 **	-0.49 ^#^	-0.05
55-64	23.6	28.7	28.8	24.9	0.96 ^#^	-0.48 **	0.02
65 and more	26.3	27.8	27.3	24.8	0.57	-0.34	-0.02
Schooling (years of study)							
0-8	16.9	20.2	20.1	17.1	0.69 **	-0.41 **	0.03
9-11	19.6	22.5	23.2	19.5	0.71 **	-0.65 **	-0.06
12 and more	27.1	31.9	34.6	27.2	0.77 **	-0.74 **	0.00
Total	20.0	24.1	25.2	21.4	0.81 **	-0.52 ^#^	0.11

* Corresponding to the Prais-Winsten regression coefficient of the indicator value over the survey year;

** p < 0.001;

*** Linear regression was performed;

^#^ p between < 0.05 and > 0.001.

